# Meningococcal Vaccines: Current Status and Emerging Strategies

**DOI:** 10.3390/vaccines6010012

**Published:** 2018-02-25

**Authors:** Pumtiwitt C. McCarthy, Abeer Sharyan, Laleh Sheikhi Moghaddam

**Affiliations:** Department of Chemistry, Morgan State University, Baltimore, MD 21251, USA; absha3@morgan.edu (A.S.); lashe9@morgan.edu (L.S.M.)

**Keywords:** *Neisseria meningitidis*, glycoconjugate vaccines, protein-based vaccines, vaccine development

## Abstract

*Neisseria meningitidis* causes most cases of bacterial meningitis. Meningococcal meningitis is a public health burden to both developed and developing countries throughout the world. There are a number of vaccines (polysaccharide-based, glycoconjugate, protein-based and combined conjugate vaccines) that are approved to target five of the six disease-causing serogroups of the pathogen. Immunization strategies have been effective at helping to decrease the global incidence of meningococcal meningitis. Researchers continue to enhance these efforts through discovery of new antigen targets that may lead to a broadly protective vaccine and development of new methods of homogenous vaccine production. This review describes current meningococcal vaccines and discusses some recent research discoveries that may transform vaccine development against *N. meningitidis* in the future.

## 1. Introduction

*Neisseria meningitidis* is a leading cause of bacterial meningitis. According to the World Health Organization, the disease has a high mortality rate (up to 50% if left untreated) and can leave 10% of those who do survive with devastating sequelae such as deafness and loss of limbs [1]. Most cases of the disease affect children under the age of 2 and between the ages of 16–21 [2]. It is estimated that one-third of disease cases affect those 65 or older. At least 12 different *N. meningitidis* serogroups have been identified, based on the chemical composition of their polysaccharides [3]. Six of these serogroups cause disease: serogroups A, B, C, W, X and Y. While most cases of meningococcal meningitis are sporadic, outbreaks still occur. Certain serogroups predominate in specific global regions [4]. Epidemics of disease caused by *N. meningitidis* serogroup A (MenA) occur in the meningitis belt of sub-Saharan Africa as well as southeastern Asia. This region of sub-Saharan Africa includes 22 countries and extends from Ethiopia to Senegal. Serogroups B and C (MenB, MenC) are responsible for most disease in Europe and North America. Disease caused by serogroup W (MenW) is common in parts of Africa and South America. It is responsible for an epidemic that occurred during the Hajj pilgrimage to Mecca nearly two decades ago. *N. meningitidis* serogroup Y (MenY) has been increasing in incidence in North America and Europe. Finally, serogroup X (MenX) is increasingly being reported in regions of Africa. Immunization strategies against those serogroups for which there are vaccines (there is currently no vaccine against serogroup X) have been crucial in helping to decrease the incidence of meningococcal meningitis [4]. This review aims to provide information on the currently licensed meningococcal vaccines and discuss some recent research discoveries that may help improve meningococcal vaccine production in the future. 

## 2. Current Vaccines against *Neisseria meningitidis*

### 2.1. Polysaccharide-Based Vaccines

There are effective vaccines for five of the six disease-causing serogroups of *Neisseria meningitidis* (A, B, C, W and Y). There are polysaccharide-based and glycoconjugate vaccines for serogroup A, C, W and Y [5]. Serogroup B is targeted by a protein-based vaccine [6]. The currently administered polysaccharide-based vaccines are quadrivalent containing capsular polysaccharide from serogroups A, C, W and Y. Monovalent (targeting serogroups A and C) and trivalent (targeting serogroups A, C and W) vaccines are no longer used. Once quadrivalent vaccines were licensed, these were administered instead of monovalent and trivalent. Mencevax (GlaxoSmithKline, Belgium) is licensed for use in Europe while Menomune (Sanofi Pasteur, Swiftwater, PA, USA) is licensed for use in the United States and Canada. Polysaccharide vaccines are composed of purified capsular polysaccharides obtained directly from the particular serogroup of the pathogen. Polysaccharide vaccines are primarily used in cases of epidemics and outbreaks [7]. A short-lived, T cell-independent immune response is generated from immunization with this class of vaccines. Conjugate vaccines elicit longer-lasting immune responses [8]. As a result, this is the major class of vaccine used to combat *N. meningitidis* serogroups A, C, W and Y [1].

### 2.2. Glycoconjugate Vaccines

Carbohydrate-based glycoconjugate vaccines use microbial capsular sugars covalently linked to a carrier protein [9]. After isolation of purified meningococcal capsular polysaccharide, it is subjected to acid hydrolysis to obtain smaller oligosaccharide fragments [10]. The resulting products are separated using chromatographic methods to obtain a particular size population for the intended vaccine. Three major types of carrier proteins have been used in vaccines against *Neisseria meningitidis*: diphtheria toxoid (DT), cross-reacting material of diphtheria toxoid with an amino acid 197 substitution which renders it inactive (CRM197), and tetanus toxoid (TT) [11]. All of these carrier proteins are inactivated forms of protein toxins from the bacterial pathogens. *Corynebacterium diphtheriae* is the source of DT and CRM197 while *Clostridium tetani* is the source of TT. The carrier proteins are crucial to inducing B cells and T cell-dependent immune responses leading to immune memory. To bring the oligosaccharides and protein together to make the vaccine, both are chemically modified to contain complementary groups that are crosslinked under proper conditions [10]. One disadvantage of this type of coupling is the resulting heterogeneity. Recent research has sought ways to make vaccine production more homogenous (discussed later). Current meningococcal conjugate vaccines are available in monovalent, quadrivalent and combination forms. These vaccines target specific age groups based on epidemiological data on disease incidence and efficacy is confirmed through clinical testing [12].

#### 2.2.1. Monovalent Conjugate Vaccines

There are currenty three monovalent conjugate vaccines licensed for *N. meningitidis* serogroup C and one monovalent vaccine against serogroup A. Two of the serogroup C vaccines (Meningtec from Pfizer (New York, NY, USA) and Menjugate from GlaxoSmithKline (Brentford, UK) use CRM197 as a carrier protein, while the other (NeisVac-C by Pfizer, Kirkland, Canada) uses TT. All three vaccines are effective in infants 2 months and younger [2]. A low-cost monovalent serogroup A vaccine with TT as carrier protein (MenAfriVac by Serum Institute of India) was developed for the meningitis belt of sub-Saharan Africa. This conjugate vaccine was produced through a unique collaboration between industry and government partners specifically the U.S. Food and Drug Administration, the Bill and Melinda Gates Foundation-funded Meningitis Vaccine Project/PATH foundation, the World Health Organization and the Serum Institute of India [13,14]. This vaccine has a broader targeted age group of ages 1–29 years old [13].

#### 2.2.2. Quadrivalent Conjugate Vaccines

Conjugate vaccines containing capsular sugars from four serogroups naturally provides broader coverage than the monovalent vaccines. They also, for the most part, cover a wider range of age groups. There are three licensed quadrivalent vaccines and each of the three carrier proteins are represented [15,16,17]. Menveo (GlaxoSmithKline, Brentford, UK) contains CRM197 as a carrier protein. Different formulations of the vaccine are effective for ages 2–23 months, 2–10 years and 11–55 years. Menactra (Sanofi-Pasteur) is a conjugate vaccine of DT with a similar age group coverage (9–23 months, 2–10 years and 11–55 years). Nimenrix (Pfizer, New York, NY, USA) is a conjugate containing TT. This particular vaccine has a narrower age range (12 months or younger). GlaxoSmithKline produces combination conjugate vaccines (described below) with broader effective age ranges.

#### 2.2.3. Combined Conjugate Vaccines

MenHibrix (Hib-MenCY-TT) and Menitorix (Hib-MenC-TT) are conjugate vaccines that are protective against serogroups of certain *N. meningitidis* serogroups and *Hemophilus influenza* b (Hib) [18]. Hib is a Gram-negative bacteria that causes pneumonia and meningitis in children under the age of five [19]. It is the first target for which a successful conjugate vaccine was developed for [20]. MenHibrix and Menitorix contain polyribosylribitol phosphate which is a major component of the capsule of *Haemophilus influenzae* b (Hib). MenHibrix targets ages 6 weeks to 18 months old. There is a two vaccine dose for Menitorix. The first dose is effective for ages 6 weeks to 12 months and the second dose is effective for ages 12 months to 2 years. 

### 2.3. Outer Membrane Vesicle-Based and Protein-Based Vaccines

Glycoconjugate vaccine strategies against serogroup B have not been pursued aggressively due to self-antigen concerns. Capsular polysaccharide from this serogroup is comprised of *α*,2-8 linked sialic acid, the same linkage of polysialic acid found on the mammalian neural cell adhesion molecule [21]. Glycoconjugates using modified sialic acid, N-propionylated sialic acid, were used in some clinical studies but those have not advanced to the licensing stage [22,23,24,25]. The first non-glycan-based vaccine against *Neisseria meningitidis* serogroup B was an outer membrane vesicle-based (OMV) vaccine licensed in Cuba [26]. OMVs are naturally occurring vesicles released by Gram negative bacteria. They contain phospholipids, lipooligosaccharides, and membrane proteins. All of those components alone can be antigens that are recognized by host antibodies. OMV vaccines can act as a self-adjuvant. VA-MENGOC-BC (Finlay Institute, Havana, Cuba) was first licensed for use in Cuba in 1987 [21]. It is comprised of OMV from a strain of the bacteria that was responsible for an epidemic in that nation. It is also contains polysaccharide from serogroup C and is therefore protective against both serogroups.

Two other OMV/protein-based vaccines targeting serogroup B have also been introduced. Protein targets for serogroup B were discovered using the concept of reverse vaccinology for the first time [27,28]. Reverse vaccinology essentially starts with a genomic search for potential antigens and the use of recombinant DNA technology to produce and test these antigens for suitability [29]. This circumvents the need to grow a specific pathogen to obtain antigens. This technology has led to licensing of Bexsero (GlaxoSmithKline, Verona, Italy) and Trumenba (Wyeth, Philadelphia, PA, USA) [6]. Bexsero, contains OMV from NZ98/254 (an outbreak-specific strain), rNHBA (a recombinant *Neisseria* heparin binding antigen) fusion protein, rNadA (recombinant *Neisseria* adhesin A), rfHbp (a recombinant complement factor H binding protein). Trumenba, on the other hand, is composed of two lipidated antigenic variants of rfHbp factors.

## 3. Emerging Methods of Vaccine Production

### 3.1. Chemical and Chemoenzymatic Synthesis of Oligosaccharides

Research efforts have evolved toward production of homogeneous glycoconjugate vaccines against *N. meningitidis* and other bacterial pathogens (recently reviewed here [30]). Current manufacturing methods for meningococcal glycoconjugate vaccines involve attachment of carbohydrate fragments to modified carrier proteins. These methods can suffer from issues such as exposure to pathogenic bacteria and low conjugation ratios [31]. Production of homogeneous meningococcal glycoconjugates are advantageous because they can circumvent such issues. This should allow for better assessment of the relationship between vaccine structure and immune response generated. In this vein, meningococcal carbohydrate antigens have been produced by chemical or chemoenzymatic synthesis. These routes are superior to isolation from the bacteria because there is no interaction with pathogenic materials and there can be better control of the carbohydrate produced. The typical method for obtaining vaccine capsular oligosaccharides for conjugation is acid hydrolysis of the isolated polysaccharide and sizing using chromatography. In complete chemical synthesis, carbohydrate chemists can adjust their chemical schemes to reach their targeted length. Chemoenzymatic synthesis requires optimization of glycosyltransferase chemical properties (i.e., by genetic mutation) and particular reaction conditions to obtain a desired target population of products. Additionally, both methods may allow for synthesis of products that mimic carbohydrate structure that can then be tested for immunoreactivity [32]. Oligosaccharides produced from chemical or chemoenzymatic methods are then conjugated to carrier proteins to produce glycoconjugate vaccine candidates. These candidates are used to immunize mice and the antibody titers are assessed for reactivity against the specific carbohydrate serogroup. Antibodies are also evaluated for their ability to kill the bacterial pathogen of interest. Activity in the serum bactericidal antibody (SBA) assay is considered to be a correlate of immune protection [33].

**Figure 1:**
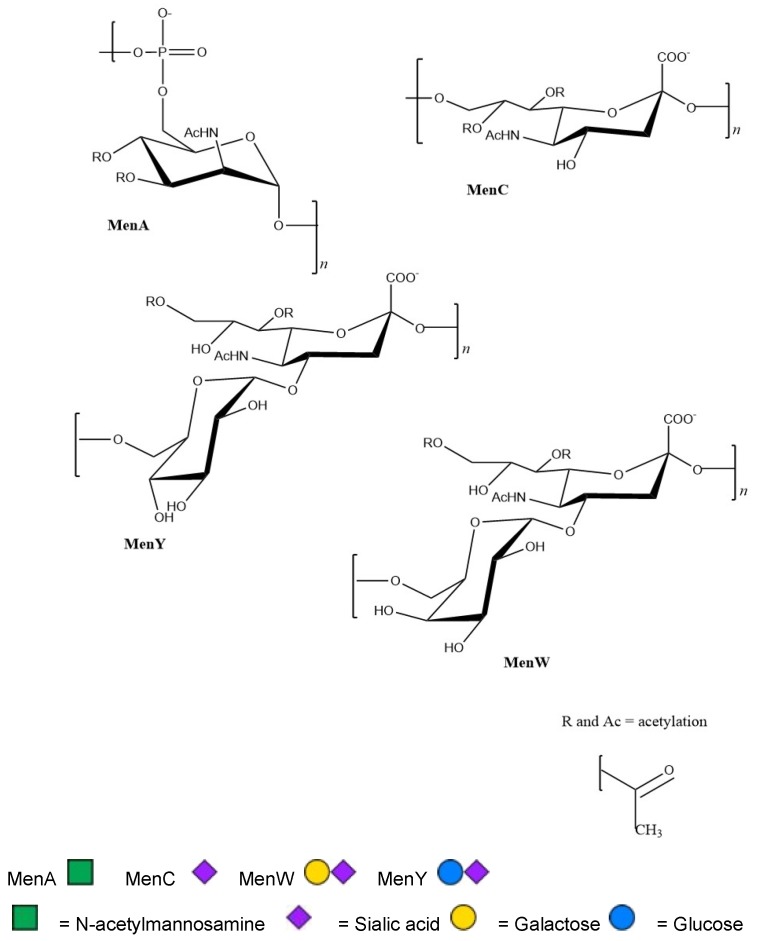
Chemical structures of monosaccharide units of meningococcal polysaccharides for which there are currently glycoconjugate vaccines. Sites of potential O-acetylation is indicated by R groups. The monosaccharide symbols follow the Symbol Nomenclature for Glycans (SNFG) according to [49].

There are a few published studies where meningococcal oligosaccharides were chemically synthesized and conjugated to a carrier protein for immunization. Chemical structures of the monosaccharide units of meningococcal polysaccharides for which there are currently glycoconjugate vaccines are given in Figure 1. The Wu group synthesized different chain lengths (degrees of polymerization, DP) of serogroup W capsule oligosaccharides. The serogroup W capsular polysaccharide contains repeating units of galactose and sialic acid. Each unit of galactose and sialic acid are linked together through an α-glycosidic linkage between carbon 1 of galactose and carbon 4 of sialic acid. The units are linked to one another through an α-glycosidic linkage carbon 2 of sialic acid and carbon 6 of galactose. Researchers from the Wu group chemically synthesized different oligosaccharides containg 1 galactose-sialic acid unit (DP2), 2 repeating units (DP4), 3 repeating units (DP6), 4 repeating units (DP8) and 5 repeating units (DP10) [34]. All of these were attached to carrier protein and used to immunize mice. Serum bactericidal antibodies were raised upon immunization with vaccine candidates containing DP4-DP10 while this wasn’t seen for DP2. These results suggest that 2 repeating units are the minimum unit required to obtain immunogenicity. Similarly, the Misra group synthesized an oligosaccharide that contained 4 units of α,1-6 linked, *N*-acetyl-3-*O*-acetyl-D-mannosamine [35]. This is the monomer unit of serogroup A capsular polysaccharide. When conjugated to TT as a carrier protein, researchers obtained antibodies capable of killing *N. meningitidis* serogroup A after immunization. The Guo group successfully performed chemical synthesis of sialic acid oligomers up to DP2-DP5 containing α-2,9 linked sialic acid. These were conjugated to two proteins (keyhole limpet hemocyanin and human serum albumin) and used to immunize mice [36]. Resulting antibodies were able to bind to *N. meningitidis* serogroup C bacteria suggesting recognition of the polysaccharide antigen in vivo.

The field of chemoenzymatic synthesis of *Neisseria meningitidis* oligosaccharides is where most recent research efforts have been focused. At this point in time, all of the glycosyltransferases responsible for synthesis of the capsular polysaccharides in disease-causing serogroups have been expressed in recombinant form [37,38,39,40,41,42,43,44]. The Vann group used modified acceptors to produce oligosaccharides from *N. meningitidis* serogroup C that were conjugated to the Hc fragment of TT using site-specific chemistry [45,46]. Mice were immunized with vaccine candidates and the antibodies produced were immunoreactive with serogroup C polysaccharide. Additionally, chemoenzymatic synthesis of potential vaccine components has been performed using *Neisseria* serogroups A, X, W and Y [42,43,44,47]. Recently, the Gerardy-Schann group has made significant advances in this regard. Her group has produced a conjugate vaccine using a recombinant form of the serogroup X capsule polymerase. Enzymatically-produced oligosaccharides were produced and conjugated to CRM197 using novel conjugation chemistry. The antibodies produced from immunization were found to be active in a serum bactericidal assay. In very recent work, her laboratory has optimized a solid-phase method with immobilized glycosyltransferases to produce oligosaccharides for serogroup A and X [48]. Using genetic engineering, the enzymes were optimized to produce products of a particular population of oligosaccharide chain lengths.

### 3.2. New Potential Carrier Proteins

There are three carrier proteins, as described above, that have been used in *Neisseria meningitidis* glycoconjugate vaccines. Two other carriers have been used in other glycoconjugate vaccines [9]. The outer membrane protein complex of MenB has been used in the Hib conjugate vaccine. Protein D from non-typeable *Haemophilus influenzae* has been used in a multivalent pneumococcal vaccine. A recent study investigated 28 potential carrier proteins from different types of bacteria [50]. These proteins were conjugated to a model polysaccharide and of those, eight were selected as potential carriers for *N. meningitidis*. Of those, four were found to elicit antibodies in mice that were immunoreactive against MenC and one was found to elicit antibodies against MenA, MenC, MenW, MenY and MenX. This carrier protein obtained from *Streptococcus pneumoniae* could be further optimized as a new carrier protein.

### 3.3. Advances in Lipopolysaccharides and Outer Membrane Vesicles as Vaccine Targets

A broadly protective *Neisseria* vaccine would greatly advance the fight against meningitis. Serogroup-specific vaccines are the only type of vaccines currently available against *Neisseria meningitidis*. Vaccines with broad protection could target all serogroups by containing an antigen that is shared among them. Common proposed targets have been lipopolysaccharide and outer membrane vesicles. Lipopolysaccharide, also known as LPS or endotoxin, is a lipid and carbohydrate containing molecule anchored in the outer membrane of Gram negative bacteria. It is considered to be a virulence factor in the disease. Lipopolysaccharide contains three components: Lipid A, core oligosaccharides and O-antigen polysaccharide. *Neisseria meningitidis* contains lipooligosaccharides which contain only Lipid A and core oligosaccharides. These structures are common to all *Neisseria* species so lipooligosaccharides may be a useful target for the development of a broad vaccine [51]. One potential candidate for exploration comes from the work of Seeberger’s group [52]. These researchers chemically synthesized a tetrasaccharide from the core oligosaccharide, conjugated it to a carrier protein and assessed the antibody response generated. This work revealed a key tetrasaccharide as a candidate for further study.

Outer membrane vesicles (OMVs) have been investigated for many years for vaccine development [53]. Recent work has sought to make OMVs more tractable as potential candidates by decreasing the toxicity of the LPS it contains. Deletion of specific genes of the LPS biosynthetic pathway (such as *lpxL1*) has led to production of OMVs with drastically reduced toxicity [54,55,56] . Additionally, genetic alterations have been explored to increase OMV production [56]. These have been explored as new candidates in pre-clinical trials of OMV-based vaccine candidates.

### 3.4. Novel Protein Targets

With the successful introduction of the two protein-based vaccines for *N. meningitidis* serogroup B, alternate protein targets have also been investigated. Porin protein A and porin protein B have long been proposed as targets for serogroup B [57,58]. These proteins are essential for pathogenesis and can occur in different ratios in different strains. Recent work by the Bash group has indicated some key elements of the porin protein structure that may serve as the minimum length required to obtain immunogenicity [59]. Other novel protein targets have been discovered using genomic, transcriptomic and proteomic approaches (reviewed here [54]). These targets are usually putative proteins believed to be expressed on the surface of the bacteria. One protein, macrophage infectivity potentiator protein has been investigated as a potential new target for serogroup B because it is conserved among strains [60,61]. The recombinant form of the protein was obtained and a liposome bound form of the protein was more immunogenic than a control and alum adjuvanted delivery of the protein [62]. The Christodoulides research group has recently investigated an adhesin protein and an ABC transporter protein as potential protein targets for a broadly protective vaccine [63,64]. Bacterial adhesins are essential proteins to facilitate host-microbe binding. Transporter proteins of the ABC type couple the energy release of ATP hydrolysis to small molecule transport across the cell membrane. The group determined that a putative *N. meningitidis* serogroup B amino acid ABC transporter, NMB1612 (in the presence of adjuvant or in liposomes), can successfully elicit bactericidal antibodies. These antibodies can also target different disease-causing strains. A similar trend was seen with adhesin proteins. Other investigated targets that are predicted to be cytoplasmic proteins in high levels in outer membrane vesicles are: NMB0928 and NMB0088 [65,66]; recombinant lipidated transferrin protein [67], RmpM protein [68,69], and heat-shock/chaperonin 60 [70](which may serve as candidate for broad protection).

### 3.5. Nanoparticulate Vaccine Delivery

Nanoparticulates are small nanoscale spherical compounds that have antigens either covalently attached, embedded non-covalently to the surface or fully encapsulated by the particulate (reviewed here [71]). All of these forms are meant to mimic how a pathogen presents antigen to a host. The types that have been explored for general vaccine use are virus like particles, liposomes, immune stimulating complexes, polymeric nanoparticles, nondegradable nanoparticles. Most alternate delivery studies for *N. meningitidis* have focused on liposomes [62,67,68,72,73,74,75]. Liposomes contain a lipid bilayer or double lipid bilayer. The interior of the liposome provides an aqueous compartment for the antigen.

The Mekalanos group has done work with components of the bacterial type IV secretion systems (T6SS) [76]. These systems are responsible for moving proteins between effector cells and target cells. Cytoplasmic sheaths containing heterodimers of VipA-VipB proteins from T6SS were recombinantly expressed and fused to the *N. meningitidis* serogroup B protein antigen fHbp. These fHbp-fused sheaths were used to immunize mice. The researchers observed the highest immune response with fHbp-fused sheaths. This response was greater than antibody levels obtained from mice immunized with free sheaths or free fHbp. Additionally, the fused sheaths produced a greater response than mice immunized with free fHbp and free sheaths combined in one injection. Thus, fusion of antigens to these VipA-VipB sheaths may offer a new route of nanoparticulate vaccine delivery.

Recent work from researchers at GlaxoSmithKline has sought to transform delivery of the vaccine from an intramuscular injection to delivery through the dermis of the skin [77]. One advantage of this route is that the skin has more antigen presenting cells than muscle [78]. A new formulation of a serogroup C vaccine was prepared for intradermal delivery using an immune stimulating complex emulsion. This produced a higher immune response than a comparable intramuscular injectible vaccine. Future work will apply the same techniques to other serogroups to further assess whether this route of delivery is a viable option. 

## 4. Perspectives and Future Outlook

Targeted vaccines have been effective at reducing the public health burden of meningococcal meningitis across many regions of the globe. Glycoconjugate and now protein/OMV-based vaccines target most serogroups of *N. meningitidis* that cause disease. The work of basic researchers and clinical researchers have helped advance the field. This review has sought to describe the current meningococcal vaccines and new approaches for the future. Recent protein-based vaccines now target *N. meningitidis* serogroup B. A suitable strategy for this serogroup has been elusive for so long. Future work in protein-based vaccine discovery seeks to discover broadly protective protein targets. Lipooligosaccharides and outer membrane vesicles are also under investigation for new targets for a broadly protective vaccine. A number of glycoconjugate vaccines against *N. meningitidis* A, C, W and Y using conventional methods of vaccine production exist. New discoveries aim to create well-defined homogeneous vaccines for which the carbohydrate antigen structure-immunogenicity relationship can be better determined. These strategies use chemical and/or enzymatic methods to produce carbohydrates. However, to date there is only one glycoconjugate vaccine on the market containing a fully synthetic carbohydrate antigen. Quimi-Hib was licensed in Cuba in 2004 and targets *Haemophilus influenza* b [79,80]. Progress in licensing of additional synthetic glycoconjugate vaccines has been slow.

In the case of meningococcal vaccines, one potential reason may lie in the difficulties of chemical and/or enzymatic synthesis. Many of the disease-causing polysaccharides (from serogroups A, B, C, W and Y) are O-acetylated at specific positions in vivo (Figure 1). These O-acetyl groups may act to increase virulence and mask the antigen from immune cells [81]. This site-specific O-acetylation is challenging to recapitulate by strictly chemical methods (although possible, see Section 3.1) Enzymatic methods to produce O-acetylated polysaccharides require recombinant production of the proper O-acetyltransferases and capsule producing enzymes [82,83,84]. Enzymatic synthesis also requires significant time in the isolation of recombinant enzymes to sufficient purity and activity. Recently a one-pot chemoenzymatic synthetic approach was proposed to obtain O-acetylated sialic acid monomers however this has not been applied to oligomers [85]. Another barrier to success may be the iterative process that needs to take place in order to determine the proper epitope that can produce serogroup-specific bactericidal antibodies [86]. This time-consuming process involves (as described in Section 3.1) synthesis of various oligomer lengths, attachment to a carrier protein and assessment of the immune response generated. Recent advances have been made in improving the sensitivity and reliability of carbohydrate microarray technology so this method could be applied to *N. meningitidis* vaccine development in the future [87]. Microarrays could dramatically streamline the discovery process because carbohydrate antigens would first be chemically attached to a microarray plate and then tested for immune response using clinically derived serogroup-specific antibodies in a high-throughput format. Lastly, while chemical and enzymatic synthesis of meningococcal carbohydrate antigens have shown promise on the research-scale, these methods still need to be optimized to manufacturing-scale levels. Future advances built upon current foundations in solid-phase chemical synthesis, enzyme production and chemoenzymatic synthesis are needed to help drive vaccine development forward. 

Carrier proteins are also critical components of homogenous vaccines. As discussed, recent studies have investigated novel carrier proteins to produce vaccine candidates. However these new proteins will require a substantial amount of laboratory and clinical studies to establish their safety and efficacy before use in glycoconjugate vaccines. In the short term, advanced mass spectrometry techniques can be used to specifically pinpoint sites where carrier proteins have been modified and carbohydrates have been conjugated [46,88,89]. New applications of site-specific conjugation methods to already established carrier proteins will also help facilitate production of homogenous vaccines [46]. In addition, further characterization of the effects inactivation has on a carrier protein will help provide more insight on the final structure in glycoconjugate vaccines [90]. In closing, there have been many recent scientific research advances that will positively impact meningococcal vaccine development so that new safe and effective vaccines can be brought to market faster. Most pressing for the immediate future, is development of a vaccine for *N. meningitidis* serogroup X as the prevalence of this serogroup is increasing [91] .

## 5. Conclusions 

Vaccination strategies against meningococcal meningitis include polysaccharide, glycoconjugate, combined conjugate and protein/OMV based vaccines. These vaccines have been proven to be safe and effective against *N. meningitidis* serogroups A, B, C, W and Y. Glycoconjugate vaccines of the future will likely use approaches such as chemical/chemoenzymatic synthesis, advanced carrier protein characterization and site-specific conjugation chemistry to obtain homogeneous vaccines. These approaches are already underway in the development of a glycoconjugate vaccine for *N. meningitidis* serogroup X for which there is currently no protective vaccine.
